# Maternal substance used during labor and neonatal outcome

**DOI:** 10.1192/j.eurpsy.2024.726

**Published:** 2024-08-27

**Authors:** J. Na Bangxang, D. Sudjai, V. Arthayukti, S. Cheepo, K. Pipatjarussakul

**Affiliations:** ^1^Psychiatry; ^2^Obstetrics and gynecology, Rajavithi Hospital, Bangkok, Thailand

## Abstract

**Introduction:**

Substance use during pregnancy has become challenging clinical issue. Substance affects the brain, causing an addictive lifestyle. In pregnant women could lead more harm to neonatal life.

**Objectives:**

This study investigates the neonatal outcome of substance use and associated factors.

**Methods:**

A cross-sectional study was designed. Data were collected from pregnant women who used substance during labor and refer to rehabilitative consultation between 2017-2020. Neonate data were collected from perinatal care. Chi-square test and Fisher exact test were performed to analyze associated factors. A p-value less than 0.05 is considered significantly.

**Results:**

162 participants were included in this study. Mean age was 27.37±6.46 years. Mean age at first substance used was 21.93±6.52years. No antenatal care was found 45.7%. Methamphetamine was the most used during the first use (67.9%) and latest used (72.2%). Average birth weight was 2,734.97±617.51 gram. Gestational age at birth was 36.75±2.83 week. Average head circumference was 32.81±1.39 centimeters. Average femur length was 47.77±2.17 centimeters. Apgar score > 7 at 1 minute and 5 minute was found 94.4% and 97.2% . Neonatal complications were preterm labor (34.6%), low birth weight (25.3%), small for gestational age (19.8%), premature rupture of membranes (4.9%), and stillbirth (3.7%). No antenatal care (p=0.048), no antenatal care and birth before admission (p=0.023), a cesarean delivery (p=0.024), and gestational age more than 37 weeks (p<0.001) were associated with neonatal outcome in maternal with substance used during labor. Using amphetamine as the first substance related to neonatal complication (p=0.028).

**Conclusions:**

Preterm labor, low birth weight and small gestational age are the most found as neonatal complications in maternal substance used during labor. No antenatal care was related with neonatal complications in this group.

Therefore, an integrated system for the assessment of substances used in a pregnant woman and the system to reach out women who used substance and pregnant access to antenatal care should be established. Evaluation and rehabilitation are the interventions that should be done as soon as possible as primaray, secondary intervention.

**Disclosure of Interest:**

None Declared

